# Nanohydroxyapatite and Bioactive Glass Composites in Bone Regeneration: A Systematic Review

**DOI:** 10.7759/cureus.100455

**Published:** 2025-12-30

**Authors:** Shrinit Babel, Sunit Babel, Syed R Peeran, Binu Pratap

**Affiliations:** 1 Morsani College of Medicine, University of South Florida, Tampa, USA; 2 Judy Genshaft Honors College, University of South Florida, Tampa, USA; 3 Neurosurgery, Christian Medical College, Vellore, IND; 4 Hand and Leprosy Reconstructive Surgery, Christian Medical College, Vellore, IND

**Keywords:** bioactive glass, bone tissue regeneration, glass nanoparticles, hydroxyapatite, systematic review

## Abstract

Bone tissue engineering is a pillar of regenerative medicine; traditional bone autografts, allografts, and metallic implants face difficulties due to donor site morbidity, risks of infection, and poor integration with the host bone. Nanohydroxyapatite (nHA) has become a popular synthetic tissue-engineered approach, although its brittleness and low fracture toughness limit its applications. This systematic review explores nHA-bioactive glass composites (HAGNs) as promising solutions for bone regeneration, analyzing their biocompatibility, osteoconductivity, and mechanical properties. This systematic review followed the Preferred Reporting Items for Systematic Reviews and Meta-Analyses (PRISMA) guidelines. A comprehensive search strategy was employed across multiple databases from January 2000 to February 2024, using MeSH terms related to hydroxyapatite (HA), glass nanoparticles, and bone regeneration. Eligible studies included peer-reviewed articles reporting outcomes relevant to bone healing or tissue engineering using HA glass nanoparticles. The search yielded 57 records, with 15 studies meeting the inclusion criteria after screening and selection. These studies explored various fabrication methods and consolidation techniques for nHA-based scaffolds, highlighting their improved biocompatibility, osteoconductivity, and potential for bone regeneration. Limitations involved long drying periods, the need for specific consolidation conditions, and more studies to confirm long-term performance. Findings underscored the importance of preserving key properties of nHA, such as low crystallinity and nanoscale dimensions, to mimic natural bone tissue effectively. nHA and bioactive glass composites show significant potential in bone tissue engineering. They offer the combination of biocompatibility, osteoconductivity, and mechanical strength necessary for effective bone regeneration. Further research is needed to facilitate the clinical translation of these biomaterials.

## Introduction and background

With the paramount demand for treating bone defects and diseases, bone tissue engineering has become a significant part of regenerative medicine [1]. Traditional bone grafting techniques, such as autografts, allografts, and xenografts, have been associated with limitations, including limited supply, donor-site morbidity, risk of disease transmission, and immune rejection [1,2]. These several limitations, therefore, paved the way for the development of synthetic bone substitutes, among which hydroxyapatite (HA) can be taken as a lead material due to its excellent biocompatibility and similarity to the mineral phase of human bone. This systematic review will focus on the properties and applications of HA glass nanoparticles in bone tissue regeneration, covering their potential to revolutionize treatments in bone applications.

HA (Ca_10_(PO_4_)_6_(OH)_2_) (HAp) is a naturally occurring calcium phosphate that forms the main mineral component of the bone and teeth. Its structure and chemical composition are similar to those of the natural bone, supporting new bone formation by attachment, proliferation, and differentiation of osteoblasts [3,4]. However, early macro-scale HAp implants were limited by intrinsic brittleness and low bioactivity. These limitations drove the development of nanohydroxyapatite (nHA), which is engineered to mimic the inherent nanoscale dimensions, low crystallinity, and non-stoichiometric nature of biological apatite [5]. The increased surface area and nanoscale topology of nHA enhance protein adsorption, cell-material interactions, and osteointegration, resulting in improved bone regeneration outcomes compared to conventional HAp [6-7]. More recent improvements in scaffold design have focused on incorporating nHA into bioactive glass (BG). Silica, calcium oxide, and phosphorus oxide in bioactive glass (BG) can bond to the bone and stimulate osteogenesis [8]. The mechanical properties, bioactivity, and biocompatibility of the nHA/sodium silicate BG composites are much superior to nHA alone [9-10].

The osteoconductive properties of nHA-based scaffolds are critical for their success in bone tissue engineering. Studies have shown that these scaffolds promote attachment, proliferation, and differentiation of osteoblasts [11]. Enhanced cell proliferation was observed in MG-63 osteoblast-like cells exposed to the composite scaffold, which tended to grow with pseudopod-like extensions, demonstrating the cells' highly adhesive and proliferative nature [12]. Likewise, biocompatibility is one of the most important criteria that any biomaterial must fulfill for clinical application. In vitro cytotoxicity studies have shown that nHA scaffolds are non-cytotoxic and suitable for clinical applications [13].

Various techniques can convert nHA into a three-dimensional scaffold while preserving its desirable properties. In addition to newer scaffolds, novel fabrication methods are being integrated with bioactive glass, enabling the development of injectable, self-setting composites and broadening their potential [12-15]. The hybrid development of nHA with bioactive glass (HAGN) is being considered a novel methodology in bone tissue engineering [16]. However, the existing evidence remains fragmented, consisting mainly of isolated experimental studies without a comprehensive systematic review that integrates fabrication strategies, material composition, and biological and mechanical outcomes [16]. This systematic review aims to synthesize the current scientific literature and provide a comprehensive overview of the properties, synthesis methods, and applications of HAGNs in bone tissue regeneration.

## Review

Methods

This systematic review followed the Preferred Reporting Items for Systematic Reviews and Meta-Analyses (PRISMA) guidelines [17]. The present review aims to provide a comprehensive assessment of the clinical benefits, risks, and effectiveness of HAGNs by analyzing bone regeneration with HAGNs compared with traditional bone graft materials.

The literature search was conducted across MEDLINE, Web of Science, EMBASE, Scopus, Cochrane’s CENTRAL, and Google Scholar. We searched for articles published from January 2000 to February 2024 and also checked the bibliographies of relevant primary research papers and systematic reviews. The MeSH terms used included "Hydroxyapatite", "Glass Nanoparticles", "Bone Regeneration", "Tissue Engineering", "Bone Grafts", and "Biocompatible Materials". A sample MEDLINE search string was (“Hydroxyapatite”[MeSH Terms] OR “nanohydroxyapatite”[Title/Abstract] OR “nHA”[Title/Abstract]) AND (“bioactive glass”[Title/Abstract] OR “glass nanoparticles”[Title/Abstract]) AND (“Bone Regeneration”[MeSH Terms] OR “bone tissue engineering”[Title/Abstract]) AND (scaffold[Title/Abstract] OR composite*[Title/Abstract])*. Only the first 100 results (sorted by relevance) were screened with each search.

The eligibility criteria were guided by the Study design, Participants, Interventions, Comparisons, and Outcomes (SPICO) framework, as shown in Table 1. Particularly, we included peer-reviewed *in vivo* and *in vitro* studies in human or animal models assessing HAGNs for bone regeneration that reported measurable outcomes with a comparator or reference group. Studies were excluded if they were *in vitro-*only, unrelated to bone regeneration, not peer-reviewed, or lacked sufficient detail to understand efficacy. 

**Table 1:** PICO criteria

(S)PICO	Description
Population	Patients or experimental models undergoing bone tissue regeneration
Intervention	The application of hydroxyapatite glass nanoparticles
Comparison	Comparative studies with conventional bone graft materials or untreated controls
Outcome	Measurable outcomes related to bone healing, bone regeneration, or bone tissue engineering
Study design	Randomized clinical trials, prospective and retrospective studies, clinical trials, and observational studies

Screening and Selection

Studies evaluating the efficacy of HA glass nanoparticles in regenerating bone tissue were included. Peer-reviewed journal research on either animal models or human subjects for this particular application was sought to be relevant. Research articles were peer-reviewed to ensure the dependability and accuracy of the data. Trials featuring a comparison group, employing either conventional bone graft materials or untreated controls, were favored for determining the effectiveness of HA glass nanoparticles relative to each other. Finally, preference was given to English-language articles.

Applications of HA glass nanoparticles unrelated to bone tissue regeneration are excluded. Additionally, non-peer-reviewed publications, including conference abstracts and unpublished data, may be of lower quality and rigor and thus are outside the criteria for inclusion. In the same vein, studies whose outcomes lack direct relevance to bone regeneration or tissue engineering are not qualified for this review. Inclusion will be limited to trials with *in vivo* studies, thereby promoting the relevance and applicability of findings in clinical settings. Lastly, studies that lacked sufficient data or that employed poor methodology to the extent that the effectiveness of HA glass nanoparticles for bone regeneration could not be assessed will be excluded from the review.

Two authors (Shrinit Babel and Sunit Babel) worked in unison to search for and screen studies; this resulted in a higher kappa coefficient of 0.83, indicating a high level of agreement. A four-stage review process was followed. Stage 1 involved the exclusion of irrelevant citations, followed by stage 2, which involved thorough scrutiny of titles and abstracts to meet predefined inclusion criteria. If there was any doubt about the articles, a full content review was conducted by a second reviewer. Stage 3 involved a stringent independent review of the chosen articles by two persons, incorporating study design, outcome measures, and the quality of the references. Papers that did not meet the stringent criteria for applying a sound methodology and data credibility were not included. At Stage 4, pertinent data from identified articles that had passed earlier inclusion criteria were abstracted and critically appraised for clinical-methodological credibility. 

Data Extraction and Analysis

The primary author performed the initial data extraction with the highest level of sensitivity. Thereafter, a second inspection and editing by the co-author were conducted to ensure the best possible accuracy and completeness of the extracted data. It was all organized in a Microsoft Excel file (Microsoft Corp., Redmond, WA, USA).

Data such as authorship, year of publication, study design, and participant demographics were recorded. Other data extracted from the studies include intervention details and comparator elements. Extracted parameters included nHA/BG ratio, Ca/P ratio, porosity (%), pore diameter (μm), compressive strength (MPa), and cell viability outcomes. The outcomes from each study were extracted to include all relevant details for further analysis. Particularly, the relationship between fabrication methods and scaffold properties was analyzed using Pearson correlation matrices. Due to heterogeneity in study design, all correlation findings were interpreted as exploratory trends rather than confirmatory statistical relationships.

Assessment of Risk of Bias

The quality of the studies and their individual risk of biases were appraised using the Risk of Bias in Non-randomized Studies of Intervention (ROBINS-I) tool [18]. Two authors independently reviewed the selected studies.

Results

Search and Selection

**Figure 1:**
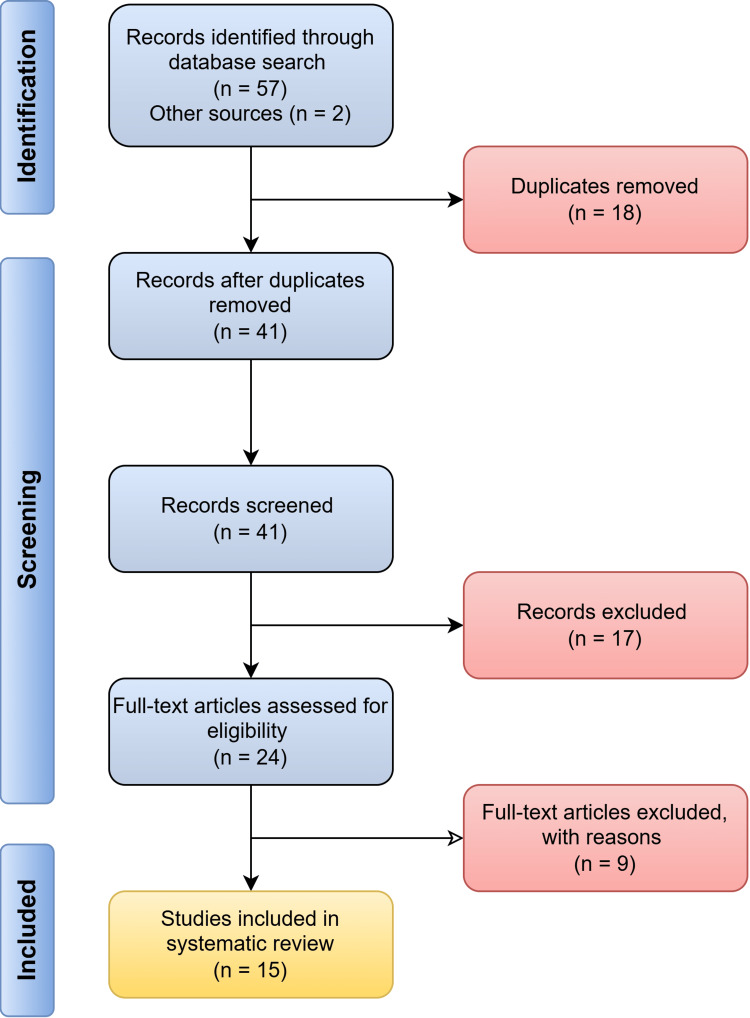
Literature search following the PRISMA flow

**Figure 2:**
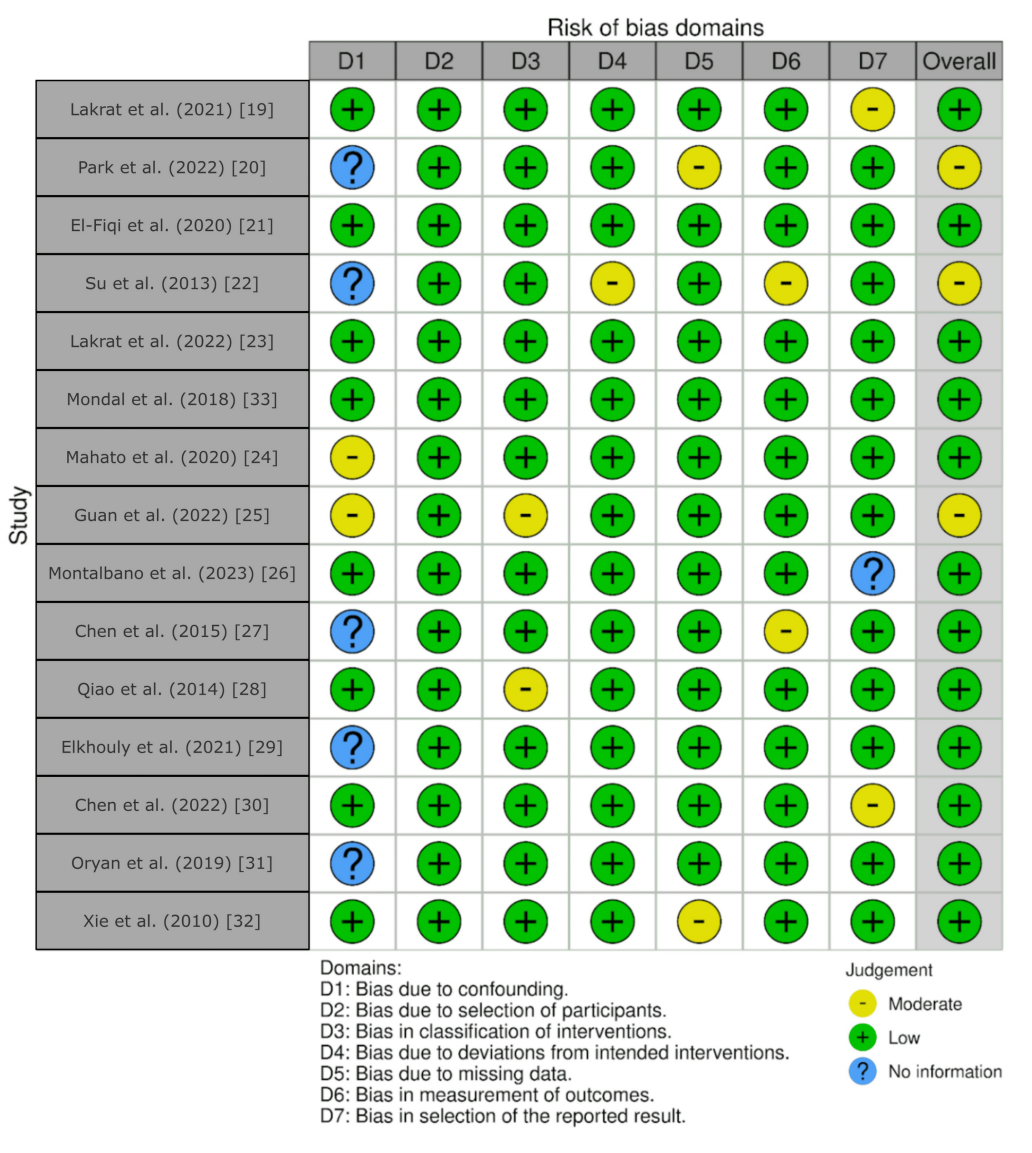
Traffic-light illustration on the risk analysis of selected studies following the ROBINS-I tool

Fifteen studies were included in the systematic review after the comprehensive search and study selection [19-33]. The details are illustrated in Figure 1. A traffic-light illustration of the risk analysis following the ROBINS-I tool is shown in Figure 2. The most common bias sources were related to confounding and reporting selection. Studies that lacked *in vivo* validation or relied primarily on *in vitro* assays were more likely to have unclear or moderate risk [25]. While most studies had low overall bias, methodological limitations in certain experimental models necessitate careful interpretation.

A tabulated summary of the articles is shown in Table 2. Eleven of the 15 studies had purely *in vitro* evaluation schemes, while three included both *in vitro *and *in vivo* validation; one study was exclusively *in vivo*. Notably, the *in vitro* studies provide insights into the mechanisms of HAGNs at a cellular level but should be interpreted in the context of evidence on efficacy and performance from *in vivo* studies.

**Table 2:** HAGN composite, consolidation methods, and outcomes per study XRD: X-ray diffraction, nHA: nanohydroxyapatite, SBF: simulated body fluid, EDS: energy-dispersive spectroscopy, FTIR: Fourier-transform infrared spectroscopy, TEM: transmission electron microscopy, FESEM: field emission scanning electron microscopy, BMSC: bone marrow-derived mesenchymal stem cell, MSC: mesenchymal stem cell, PCL: polycaprolactone, Gt: gelatin, BG: bioactive glass, OCN: osteocalcin.

Study	Design	Bioactive binder used	Consolidation method for nHA	Osteoconductive properties	Physicochemical properties
Lakrat et al. (2021) [19]	In vitro	Sodium silicate	Dehydration-drying	Enhanced proliferation of MG-63 osteoblast-like cells	High porosity (46 ± 2%), average compressive strength of 21.3 ± 2 MPa
Park et al. (2022) [20]	In vitro; ex vivo	63S bioactive glass (65 mol% SiO2, 31 mol% CaO, 4 mol% P2O5)	Wet chemical precipitation	Pseudopod-like cellular extensions on MG-63 osteoblast-like cells	Thermal stability: maximum loss of 10.73%. Crystallinity: XRD analysis indicated good phase stability, distinct crystalline peaks. Porosity: microporous structure
El-Fiqi et al. (2020) [21]	In vitro; in vivo	Surface silanized mesoporous bioactive glass	Surface silanization, collagen hybridization, Biomimetic mineralization in SBF	Significant osteogenesis in rat calvarial defects. Histological analysis indicated a higher number of osteocytes in mineralized scaffolds compared to non-mineralized scaffolds	Crystallinity: crystal size of nHA was controllable; increased from 18.59% to 22.86% in one week. Composition: EDX analysis confirmed the formation of HA with near-stoichiometric Ca/P ratios. Porosity/structure: microporous structure (100-300 μm)
Su et al. (2013) [22]	In vitro; in vivo	Bioactive glass fiber	Ethanol co-precipitation, extrusion, and injection molding	Improved adhesion, proliferation, and viability of MC3T3-E1 cells. Histological analysis showed extensive osteogenesis and integration with host bone.	Crystallinity: the nHA coating layer had high crystallinity. Composition: EDS analysis confirmed formation of HA. Structure: nHA particles maintained their nanoscale dimensions
Lakrat et al. (2022) [23]	In vitro	Sodium silicate	Dehydration-drying	Enhanced proliferation of MG-63 osteoblast-like cells	Crystallinity: the crystallinity of nHA was maintained during processing. Structure: nHA particles maintained their nanoscale dimension. Composition: HA formation with near-stoichiometric Ca/P ratios
Mondal et al. (2018) [33]	In vitro	63S bioactive glass and alumina	Wet chemical precipitation, ball milling, gel-casting	Enhanced cell proliferation, viability, and attachment on scaffold surface. Formation of pseudopod-like extensions.	Crystallinity: XRD analysis indicated distinct high-intensity peaks. Composition: EDS analysis confirmed Ca, P, Al, and Si with near-stoichiometric ratios. Thermal stability: minimal mass loss upon heating
Mahato et al. (2020) [24]	In vitro	Sodium silicate	Sol-gel method, freeze-drying	Enhanced cell proliferation and viability of MG-63 cells cultured on the scaffold glass. Enhanced ALP activity.	Crystallinity: XRD confirmed the presence of characteristic diffraction peaks. Composition: FTIR and Raman Spect showed typical vibrational modes of PO43 groups, suggesting the presence of HAp. Structure: TEM and FESEM show microporous structure with interconnected pores
Guan et al. (2022) [25]	In vitro	Mesoporous bioactive glass	Calcium-based cross-linking	Improved deposition of bone-like apatite on scaffold hydrogel surface. Enhanced alkaline phosphatase (ALP) activity in mouse BMSC	High compressive strength of 170-220 kPa, good injectability
Montalbano et al. (2023) [26]	In vitro	Mesoporous bioactive glass	Collagen hybridization, genipin cross-linking	The material supported deposition of bone matrix	Structure: stable fibrous matrix
Chen et al. (2015) [27]	In vitro	Cellulose nanocrystals and bioactive glass	Electrophoretic deposition	Rapid formation of a single hydroxyapatite layer on the coating within 0.5 days	Composition: rapid HA formation. Structure: porous structure
Qiao et al. (2014) [28]	In vitro	Polyamide 66 and glass fibers	Extrusion	Promoted osteogenic differentiation of MSCs. Alkaline phosphatase and osteocalcin expression increased over time	Structure: well-integrated without matrix cracking; strong adhesion between n-HA/PA66 matrix and glass fibers
Elkhouly et al. (2021) [29]	In vitro	Bioactive glass nanoparticles (45% silica, 25% CaO, 25% Na2O, and 5% P2O5, with particle sizes less than 10 nm​)	Electrospinning	SEM and EDX analyses revealed formation of hydroxyapatite after immersion in simulated body fluid for 14 days	Composition: FTIR showed the presence of PCL, Gt, and BG, indicating successful incorporation of all components. Structure: high surface area and total pore volume
Chen et al. (2022) [30]	In vitro	Bioactive glass + PDMS/PCL matrix	Sol-gel process, thermal casting	XRD and SEM analyses confirmed the formation of hydroxyapatite after immersion in simulated body fluid	Structure: crack-free structure with high surface roughness. Composition: EDS and XRD analyses confirmed the presence and uniform distribution of nHA
Oryan et al. (2019) [31]	In vitro; in vivo	Bioactive glass and strontium	Sol-gel process	Promoted osteogenic differentiation of BMSCs into osteoblasts. Enhanced expression of ALP, OCN, and collagen	Structure: SEM analysis showed a homogenous porous structure with interconnected pores. Composition: XRD patterns confirmed the presence of crystalline phases consistent with HA, indicating successful incorporation of nHA, Sr, and BG
Xie et al. (2010) [32]	In vivo	Bioactive glass	Sintering technology	Increased bone-implant contact over time. Higher bone volume observed in BG-nHA implants compared to HA-coated and uncoated implants. Gradient coating supported the attachment and growth of hBMSCs	Structure: SEM analysis showed that the surface of the gradient coating was composed of round-shaped crystals (100 nm diameter). Composition: XRD analysis confirmed HA crystals with high crystallinity

Composite Formation and Fabrication Techniques

**Figure 3:**
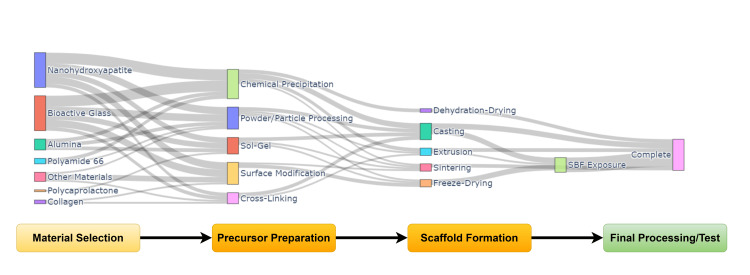
Fabrication pathways of nanohydroxyapatite-bioactive glass scaffolds across studies Image Credit: This figure was designed by the authors using Python.

Designing HAGNs is a multi-step process that integrates material selection, precursor preparation, scaffold formation, and final processing steps or testing. The fabrication pathways used across studies to develop HAGNs are illustrated in Figure 3.

All studies used BG, albeit in different variations. Most studies used mesoporous BG nanoparticles to improve apatite formation and ion release for osteogenic differentiation and bone regeneration [26]. In contrast, silica-based BG, such as 45S5 or 63S bioactive glass (SiO_2_-CaO-P_2_O_5_ system), was selected for scaffolds aimed at optimizing scaffold mineralization or controlling degradation kinetics, appropriate for sustained mechanical support in load-bearing applications [20,33].

Beyond differences in BG composition, some studies targeting fracture fixation devices selected the inclusion of E-glass or glass fibers in composite scaffolds, prioritizing mechanical strength while maintaining bioactivity [22,28]. The glass fiber-reinforced composites were hypothesized to mimic the structural resilience of cortical bone, reducing stress shielding effects offloaded by metallic implants. BG-processing techniques also varied, with some studies using sol-gel-derived BG for better nanoscale porosity, while others relied on melt-derived glass for durability [21,24].

In addition to nHA and BG, many studies also incorporated secondary materials into their scaffold. Polymers such as polyamide 66 (PA66) were frequently used in conjunction with glass fibers for composite bone plates and screw scaffolds, specifically to address the challenge of stress shielding with metallic implants [22,28]. Polycaprolactone, known for its processability and flexibility, was also added to some HAGN innovations to modulate degradation rates [29,30]. Alumina (Al_2_O_3_) was added to bio-cements in three studies for wear resistance, working synergistically with silica-based BG in load-bearing applications [20,24,33]. Collagen was integrated for its biomimetic properties [21,26].

Preparing the raw scaffold involved different techniques, with at least 5 of the 15 using over two techniques in sequence to create the scaffold. Wet chemical and ethanol precipitation were the most common methods for synthesizing the HAGN scaffold (6/15 studies). Precipitation-based approaches all induce the formation of solid particles or a phase change through chemical reactions. Some studies using precipitation-based approaches employed a sodium silicate solution rather than traditional bioactive glass as a novel consolidation method. These studies were especially focused on preserving the crucial properties of nHA and found improved cell proliferation and differentiation [19]. Sol-gel, while similar to precipitation-based approaches in that it involves a phase transition, follows a distinct pathway with supposedly better compositional control, higher purity, and lower processing temperatures. However, there can be challenges with particle aggregation. Notably, El-Fiqi et al. synthesized a HAGN through a rapid drying, ultrasound-assisted sol-gel method, enhancing the homogeneity of the mesoporous BG distribution [21]. The approach offered uniform particle dispersion and preserved the porous architecture of the scaffold for better cell infiltration. Ultrasound-assisted mixing, as well as ball milling, have been used in other studies as powder and particle processing techniques to improve the homogeneous incorporation of inorganic and organic phases and provide uniformity in particle size distribution.

Cross-linking with various reagents (e.g., acetic acid, genipin) or surface modification, such as through gradient coating, electrophoretic deposition, or surface silanization, was typically used in addition to other preparation techniques, depending on the application. For example, stainless steel or titanium has been coated with HAGN scaffolds onto implants to improve bone-implant osseointegration and osteoblast adhesion [27,32].

The scaffold composition and preparation approach often informed fabrication techniques, which also varied across studies. Dehydration-drying and freeze-drying were frequently used for preliminary stabilization and architecture preservation [19,24]. Casting techniques, such as gel casting, thermal casting, self-setting cement formation, and hydrogel casting, enabled the formation of complex scaffold/interface geometries, bridging the scaffolds for application [20,30,33].High-temperature sintering was used to modulate crystallinity, while extrusion techniques were focused on optimizing porosity [7,22]. 

After scaffold fabrication, over half of the studies (8/15) performed further scaffold post-processing using simulated body fluid (SBF) immersion. SBF is a solution that mimics the ionic composition of human plasma, so SBF immersion is widely used to promote early HA formation. Mondal et al. and Chen et al. found that SBF enabled early apatite nucleation and mineralization, with some samples demonstrating changes as quickly as three hours [30,33]. The rapid apatite deposition was especially noted in HAGNs with mesoporous BG formulations [26]. This contrasts with silica-based BG scaffolds, where there was slower but sustained apatite formation [27]. In other words, while SBF immersion allows for the induction of HA, the BG formulation and other scaffold features influence the mineralization kinetics. SBF immersion was also used as a performance measure.

**Figure 4:**
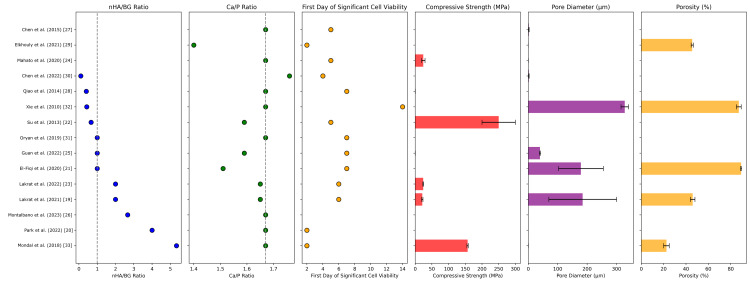
Comparative analysis of HAGN scaffolds: composition, cell viability trends, mechanical properties, and porosity

The ratios of nHA to BG (nHA/BG) and of calcium to phosphate (Ca/P) in final scaffold formulations across studies are illustrated in Figure 4. The nHA/BG ratio varied significantly across studies, due to differences in material design strategies and goals in bioactivity and mechanical performance. Studies with higher nHA content prioritized biomimetic mineralization, while those with higher BG content focused on enhanced ion release and bioactivity for regeneration [20,32]. Conversely, Ca/P ratios were typically around 1.67, closely mimicking the natural Ca/P ratio in bone HA. That being said, some studies deviated slightly to optimize degradation kinetics or improve regeneration [29].

Cell Viability, Osteoconduction, and Osteoinduction

Studies assessed toxicity, cell viability, and osteogenic potential primarily *in vitro*, using MTT or CCK-8 assays that measure the metabolic activity of osteoblasts (MCT3-E1, MG-63) or mesenchymal stem cells (MSCs) over 24-72 hours and up to 21 days [22]. Some studies also incorporated staining techniques and alkaline phosphatase (ALP) activity as measures of osteogenic differentiation. In contrast, others used material characterization techniques such as X-ray diffraction (XRD), scanning electron microscopy (SEM), and energy-dispersive spectroscopy (EDS) [24]. Osteoconduction, referring to bone cell adhesion, and osteoinduction, referring to the promotion of osteoblastic differentiation, were also analyzed. Cell infiltration and nutrient exchange served as indicators of osteoconduction, while histological findings suggestive of adequate osteoinduction included the formation of pseudopod-like extensions by osteoblast-like cells or the visualization of a greater number of osteocytes [20].

The first significant *in vitro* cell compatibility observation from each study is illustrated in Figure 4. None of the studies reported any toxicity or adverse effects with their HAGN composites, and most studies demonstrated significant cell viability 4-6 days after HAGN scaffold application. Xie et al. observed significant osteogenic activity beyond this period, with bone volume significantly increasing after 12 weeks of application [32]. This prolonged effect is likely due to their gradual ion-release approach and silica-based BG scaffold, both aimed at long-term bone remodeling. Regardless, their HAGN scaffold demonstrated compatibility early on.

In studies analyzing the effect of the scaffold *in vivo, the* absence of severe inflammation was used as a proxy for cell compatibility, while histological analyses or micro-CT imaging confirmed osteointegration and bone regeneration [22,28,32]. For example, El-Fiqi et al. and Qiao et al. found significantly higher bone formation in mineralized scaffolds compared to non-mineralized scaffolds from *in vivo* analyses [21,28]. HAGN scaffolds with interconnected porosity promoted deeper bone tissue infiltration, resulting in higher bone volume fractions and trabecular thickness over 12-24 weeks. El-Fiqi et al. found increases in bone volume fraction and trabecular thickness in micro-CT imaging with their HAGN scaffold [21].

Consistent with findings from SBF immersion analyses, silica-based BG scaffolds demonstrated long-term, sustained osteoconduction, while mesoporous BG-containing scaffolds had rapid bone-bonding potential. The HAGN scaffolds also had osteogenic and osteoinductive potential. Mahato et al. and Oryan et al. found ion release from their scaffolds, namely Ca2+, Si^4+^, and Sr^2+^, suggesting osteogenic differentiation [24,31]. The presence of Sr^2+^ ions in certain BG formulations was found to activate the Wnt/β-catenin pathway, key to osteoinduction and bone regeneration. This was confirmed with osteoblast gene expression analyses (RUNX2, OCN, OPN) and ALP activity assays.

Physicochemical Properties

There are several aspects of a composite that render it mechanically and physically stable. These include adequate thermal stability, crystallinity, porosity, and composition, as well as stress-strain mechanics. Most of the studies assessed the physicochemical properties of the HAGN grafts through their compressive strength and porosity (Figure 4). Compressive strength (σ_c_) represents the scaffold’s ability to resist deformation under compressive load (i.e., where *F* is the maximum force applied against *A*, the cross-sectional area under compression). Mondal et al. and Qiao et al. measured compressive strength using universal testing machines, where compressive forces were applied until material failure [28,33]. The compressive strength of most HAGN scaffolds was largely dependent on processing techniques and material composition. Hydrogel-based HAGN scaffolds typically had lower values (i.e., ~170 kPa), while sintered scaffolds had over 150 MPa.

Porosity and pore size are not only important for cellular infiltration but also interact with the mechanical strength characteristics of a scaffold. Most studies used mercury intrusion porosimetry and other liquid displacement methods for porosity measurement. Lakrat et al. and Park et al. found that scaffolds with a porosity between 40% and 70% enabled optimal bone ingrowth, while those with lower porosity had less bioactivity but higher mechanical strength [19,20]. Similarly, El-Fiqi et al. and Oryan et al. described that pores in the range of 100-300 μm were optimal for osteoblast attachment [21,31]. Smaller pore sizes were generally in mesoporous BG-containing scaffolds, which required additional modifications to ensure proper cell penetration and vascularization in lieu of the limited pore dimensions.

Other physicochemical parameters that were assessed by some studies related to the scaffold surface chemistry and wettability, which were typically evaluated through contact angle measurements. Chen et al. and Elkhouly et al. found that scaffold angles had better protein adsorption and subsequently, osteoblast adhesion; hydrophilic surfaces improved bioactivity [27,29]. Guan et al. and Park et al. also evaluated the injectability of the graft to gain insights into surgical ergonomics [21,27]. 

**Figure 5:**
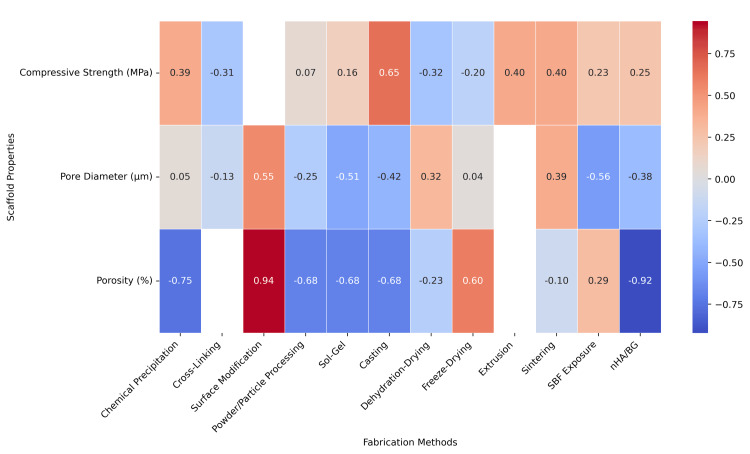
Association between fabrication methods and physicochemical scaffold properties

Across the studies, physicochemical parameters were frequently associated with scaffold fabrication techniques (Figure 5). Scaffold surface modification showed the strongest positive correlation with porosity, suggesting that techniques like gradient coatings or chemical treatments may influence scaffold architecture. Casting and sintering had moderate positive correlations with compressive strength, synonymous with prior findings in individual studies. The nHA/BG ratio had a strong negative correlation with porosity, likely due to densification of the scaffold architecture.

Discussion

Most of the innovations developed by the 15 studies highlighted the excellent biocompatibility and osteoconductivity of the HAGN scaffolds. nHA’s inertness and similarity to the bone make it a valuable material in bone engineering. However, alone, it is inherently brittle, and its low fracture toughness makes it prone to cracking under stress, such as in load-bearing applications. Also, although nHA supports bone growth, it has limited osteoinductivity, which could further enhance bone regeneration [34,35]. Another significant problem with using nHA alone or through other composites is that these composites often have weak binding due to differences in material [34,35]. Bioactive glass complements nHA by enhancing its mechanical properties, osteoconductivity, and bioactivity, and helps address the limitations of nHA and other composites. One of the key contributions of BG to HAGN scaffolds is the improvement of fracture toughness in inherently brittle nHA. Particularly, BG improves fracture resistance through ion release: across the studies, the bioactive glass promoted the release of ions such as silicon, calcium, and phosphate [36]. The release of Si^4+^ and Ca^2+^ promotes interfacial adhesion, reducing microcrack formation in the nHA matrix. Furthermore, the amorphous nature of BG provides crack-deflection mechanisms, where stress is redistributed along the interface to prevent catastrophic failure [37]. The histological analysis of several composites also demonstrated the formation of a uniform HA layer on the surface of the bioactive glass, similar to natural bone, suggesting improved bonding with natural bone. In general, the composites across the studies had significant osteoconductive properties.

That being said, the material alone did not determine the efficacy of the scaffold composite; the method of preparation and fabrication ultimately influenced its mechanical strength, porosity, and overall bioactivity. Regardless, the most suitable fabrication technique depends on its intended application, particularly whether for load-bearing or non-load-bearing sites. Casting (especially thermal casting) and sintering are more suitable for load-bearing applications, while freeze-drying and solvent-casting are better suited for nutrient diffusion in non-load-bearing scaffolds.

**Figure 6:**
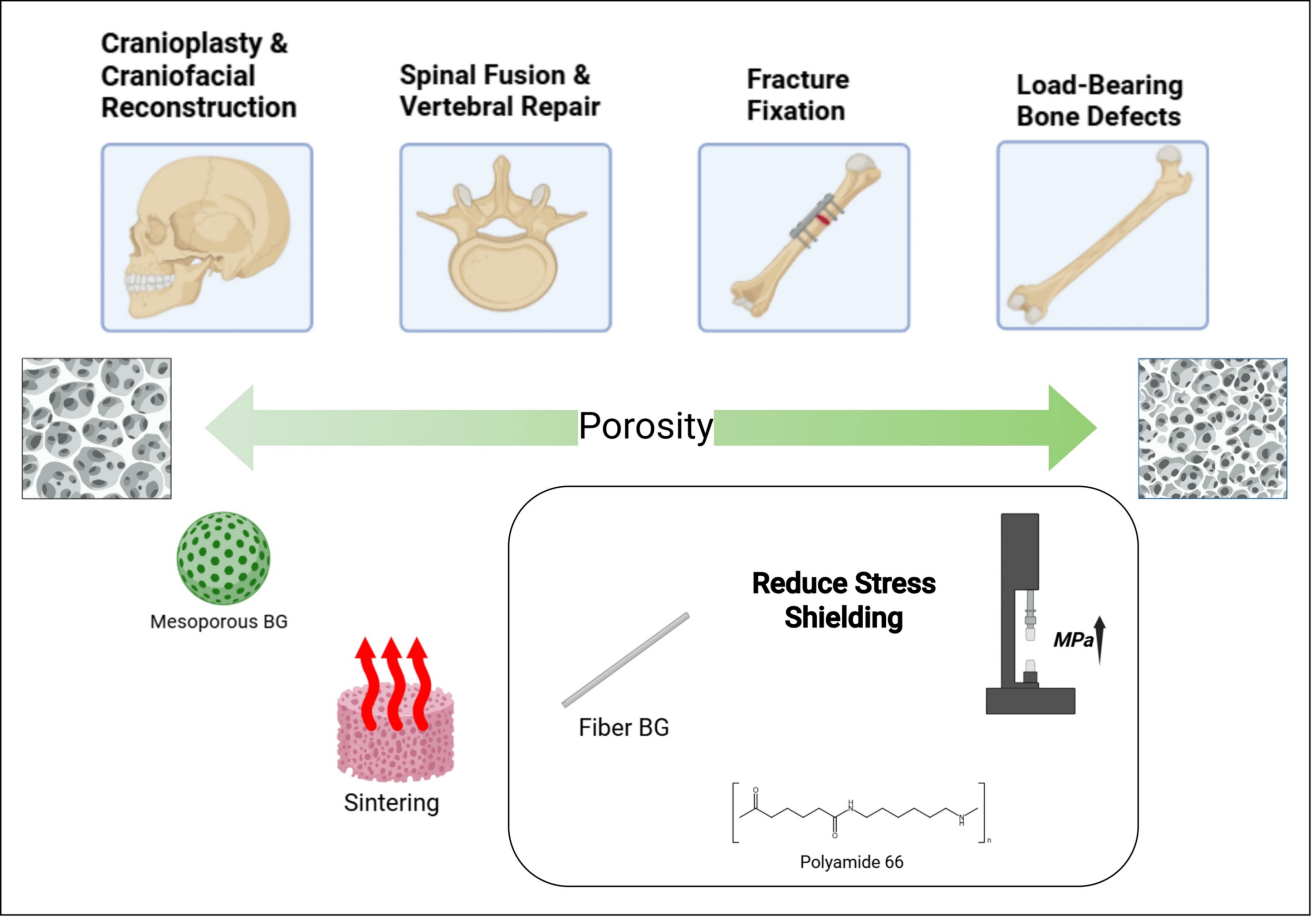
Applications of nanohydroxyapatite-bioactive glass composites in bone regeneration Image Credit: This Figure was designed by the authors using draw.io.

Considering this, HAGN scaffolds can play a role as a bioactive bone graft alternative, with applications in burr hole surgery and cranial reconstruction, spinal fusion, fracture fixation, and load-bearing bone defect repair (Figure 6). Scaffolds designed for cranioplasties or craniofacial reconstruction should prioritize high porosity for rapid bone ingrowth, making mesoporous BG composites ideal. In vertebral repair, HAGNs that provide controlled degradation can help avoid rigid fusion complications.

Implant applications require additional mechanical considerations. Higher mechanical strength is essential for anything requiring fixation plates or screws, making glass fiber-reinforced HAGN composites suitable. Sintering techniques can be used for cranioplasty implants to offset the high porosity requirements or in load-bearing bone defects, where high compressive strength is required. Stress shielding, a phenomenon where implants observe too much mechanical load, reducing physiological bone stress and leading to resorption, is a key concern in scaffold design [38]. HAGN scaffolds can mitigate stress shielding when coated onto implants by enhancing the implant-bone interaction. Polymer-reinforced BG composites can help distribute the load more evenly.

The systematic review also revealed some disadvantages of HAGN. Across the studies, fabrication emerged as a challenge. One key issue is the difficulty of achieving uniform dispersion of nHA and BG within the scaffold matrix, which can lead to heterogeneous mechanical properties. In cases where scaffold homogeneity is prioritized, powder and particle dispersion techniques, such as the ultrasound-assisted mixing and ball milling used by El-Fiqi et al., can help overcome this challenge [21]. 

The analysis of fabrication techniques on physicochemical properties, as well as independent studies, revealed an inherent trade-off between porosity and mechanical strength. Minimal porosity would prevent cell infiltration, while large porosity would jeopardize the overall scaffold. Some of the studies were able to circumvent or optimize this trade-off by using a combination of fabrication techniques. For example, sintering followed by surface modification or cross-linking was explored by some studies to improve porosity without compromising mechanical stability.

Although the bioactive glass provided flexibility in consolidating the scaffold, the fabrication methods were also time-consuming, complex, and required specific conditions. This was especially true for drying processes, as they had a long drying and mineralization period of at least 15 days [23]. One possible approach to address this is through rapid drying techniques or microwave-assisted sintering, which can potentially reduce processing time without compromising scaffold integrity [4,23].

Regardless of the scaffold innovation, it is critical to preserve the innate properties of HAp. Novel fabrication and consolidation methods introduced by the studies can offer a practical edge in terms of scalability and cost-effectiveness. Additionally, novel fabrication and consolidation methods present practical benefits in terms of scalability and cost-effectiveness. From a translational perspective, most current evidence still stems from preclinical models, with a few *in vivo* analyses. There is a lack of standardized manufacturing protocols or long-term biocompatibility data, which are necessary for clinical translation and better regulatory insight.

Limitations

The review has some limitations. Many of the included studies were conducted *in vitro* or on animal models, which restricts their direct applicability to clinical practice. The review primarily focuses on the efficacy and properties of nHA, with less emphasis on its long-term biocompatibility and stability *in vivo*. There was a lack of consistency in the fabrication techniques employed across the studies, making it challenging to find the best methods for scaffold manufacturing. Furthermore, there was limited investigation into stress-strain properties, including compressive strength. Thermal stability was also only assessed in two studies, although this aspect is key for biocompatibility and during the implant process [20,33]. More *in vivo* studies are necessary to confirm the insights drawn from this review. Nonetheless, the systematic review highlights the promising role of nHA in bone tissue regeneration and the need for further research to bridge current gaps and support clinical translation.

## Conclusions

This systematic review delves into the added osteoconductivity and bioactivity leveraged by HAGNs. Incorporating BG into nHA composites addresses the inherent limitations of traditional composites without sacrificing existing properties. The studies incorporated various consolidation and fabrication methods, including novel techniques, that helped maintain the integrity of the scaffolds. Indeed, the fabrication processes are notably complex and time-consuming, and achieving the ideal balance between nHA and BG content is also important to maintaining the composite’s mechanical strength and bioactivity. Additionally, most studies were conducted in vitro or on animal models. Despite these challenges, this systematic review opens doors to future research to understand the potential and application of HAGNs. Combining nHA with bioactive glass offers promising development for traditional bone grafts and synthetic substitutes.
